# Antimicrobial susceptibility of *Porphyromonas* spp. isolated from dogs with periodontal disease in South Korea

**DOI:** 10.3389/fvets.2025.1684907

**Published:** 2025-11-10

**Authors:** Taesoo Kim, Yi-Don Choi, Won Hur, Sang-Won Lee, Tae-Min La

**Affiliations:** 1 Microbiology Laboratory, College of Veterinary Medicine, Konkuk University, Seoul, Republic of Korea; 2 VIP Animal Medical Center KR, Seoul, Republic of Korea

**Keywords:** antimicrobial susceptibility testing, *Porphyromonas*, periodontitis, clindamycin, minimum inhibitory concentration

## Abstract

*Porphyromonas* spp. are oral anaerobes that play a key role in the pathogenesis of canine periodontal disease. Despite their clinical relevance in veterinary medicine, data on the antimicrobial susceptibility of canine *Porphyromonas* isolates remain limited. Therefore, we assessed the antimicrobial susceptibility of *Porphyromonas* spp. isolated from the subgingival plaque of dogs with periodontitis in South Korea. Fifty-eight dogs diagnosed with periodontal disease were screened for *Porphyromonas* spp., and species identification was confirmed using PCR and matrix-assisted laser desorption/ionization time-of-flight mass spectrometry. Antimicrobial susceptibility testing was performed using the broth microdilution method with the Sensititre AN02B panel. Overall, 40 isolates were recovered from 30 of the 58 dogs sampled, comprising 15 *Porphyromonas gulae*, 11 *Porphyromonas macacae*, eight *Porphyromonas gingivalis*, five *Porphyromonas gingivicanis*, and one *Porphyromonas crevioricanis*. Resistance was detected in six isolates (15%) to penicillin, three (7.5%) to imipenem, three (7.5%) to meropenem, 15 (25%) to clindamycin, and seven (17.5%) to ampicillin. No resistance was observed to ampicillin/sulbactam, amoxicillin/clavulanic acid, cefotetan, cefoxitin, metronidazole, chloramphenicol, piperacillin, tetracycline, mezlocillin, or piperacillin/tazobactam. These findings provide crucial insights into the antimicrobial susceptibility patterns of canine oral *Porphyromonas* spp. and highlight the importance of judicious antimicrobial use in veterinary dentistry.

## 1 Introduction

Periodontal disease is a chronic inflammatory condition that leads to the progressive destruction of the supporting structures of the teeth, including the gingiva, alveolar bone, cementum, and periodontal ligament. It is primarily driven by complex bacterial communities that colonize dental plaque and calculus (1, 2). Among these microorganisms, species of the genus *Porphyromonas* are frequently implicated in both the initiation and progression of periodontal disease in dogs (1, 2).

*Porphyromonas* spp. are part of the commensal oral microbiota in dogs, being detectable in both healthy and diseased states. However, their prevalence is considerably higher in diseased populations, where they often act as keystone members contributing to disease progression (3, 4). This dual presence highlights their role as part of the normal commensal flora in health and as major pathogens in periodontal disease.

*Porphyromonas* spp., along with other periodontal pathogens, typically reside within biofilms, structured microbial communities encased in a self-produced extracellular matrix. Biofilm formation markedly diminishes the efficacy of antimicrobial agents through multiple mechanisms including the physical barrier imposed by the extracellular polymeric matrix, reduced metabolic activity of bacteria in deeper biofilm layers, and the presence of persister cells (5–9). Consequently, the primary treatment for periodontal disease has traditionally relied on mechanical debridement (10, 11). However, the frequent recurrence of periodontitis following mechanical therapy alone has raised concerns about the sufficiency of this approach (12). To enhance treatment outcomes, the use of antimicrobial agents as short-term adjuncts in cases of periodontitis has become increasingly common, particularly following procedures such as scaling or extractions (10, 13). A retrospective study indicated that approximately 16.4% of canine dental procedures involved the administration of systemic or local antimicrobials (13). Beyond the oral cavity, *Porphyromonas* spp. have been associated with extraoral infections such as aspiration pneumonia, wound infections, sepsis, and hepatic abscesses, conditions that necessitate systemic antimicrobial therapy (14–20). Despite their clinical relevance in veterinary medicine, data on the antimicrobial susceptibility of canine *Porphyromonas* isolates remain limited. The fastidious nature of these bacteria and the technical challenges associated with their cultivation hinder large-scale surveillance efforts. Moreover, empirical antimicrobial use in the absence of susceptibility data may disrupt commensal microbiota and promote the emergence of resistant strains (21, 22).

In this study, we aimed to address this knowledge gap by characterizing the antimicrobial susceptibility profiles of *Porphyromonas* spp. isolated from the subgingival plaque of dogs with periodontitis in South Korea. The findings will support improved periodontal management and promote antimicrobial stewardship in veterinary practice.

## 2 Materials and methods

### 2.1 Sample collection

Dental plaque samples were collected from 58 dogs diagnosed with periodontal disease at the VIP Animal Medical Center (Seoul, South Korea) between October 2019 and August 2020. Samples were obtained from the maxillary molar region and placed in a transport medium consisting of Brucella broth supplemented with 1% yeast extract, 5% defibrinated sheep blood, 0.5 g/L cysteine, 5 μg/mL hemin, 10 μg/mL vitamin K, and 0.3% agar. Samples were immediately transported under anaerobic conditions to the Microbiology Laboratory, College of Veterinary Medicine, Konkuk University. Detailed clinical metadata were not available because the samples were provided by the collaborating veterinary hospital during routine dental procedures.

### 2.2 Bacterial isolation and identification

Samples were homogenized in 0.5 mL distilled water and streaked onto *Porphyromonas* Blood Agar (Tryptic Soy Agar supplemented with 1% yeast extract, 5% defibrinated sheep blood, 0.5 g/L cysteine, 5 μg/mL hemin, and 10 μg/mL vitamin K). Plates were incubated anaerobically at 37 °C for up to 2 weeks. All black-pigmented colonies, characteristic of *Porphyromonas* spp., were subcultured until pure isolates were obtained. Each *Porphyromonas* isolate from a given sample was stored in brain heart infusion broth containing 10% skim milk at −80 °C.

Frozen stocks were revived by streaking onto *Porphyromonas* Blood Agar and incubating anaerobically at 37 °C. Colonies were then inoculated into supplemented Brucella broth and incubated anaerobically at 37 °C for 2 weeks. Following incubation, bacterial cultures were smeared onto a matrix-assisted laser desorption/ionization time-of-flight mass spectrometry (MALDI-TOF MS) target plate. To each smear, 1.5 μL of 70% formic acid was applied and allowed to dry completely, followed by 1.5 μL of matrix solution. Once dry, the samples were analyzed using MALDI-TOF MS by NosVet Co. (South Korea).

For molecular confirmation, genomic DNA was extracted from cultured isolates using the QIAamp DNA Mini Kit (Qiagen, Hilden, Germany) according to the manufacturer’s instructions. The 16S rRNA gene was amplified by PCR using primers 8F (5′-AGA GTT TGA TCC TGG CTC AG-3′) and 1429R (5′-GGT TAC CTT GTT ACG ACT T-3′) (23, 24), in accordance with the i-Taq DNA polymerase protocol (iNtRON Biotechnology, South Korea).

The PCR conditions included an initial denaturation at 95 °C for 5 min, followed by 35 cycles of denaturation at 95 °C for 1 min, annealing at 55 °C for 30 s, and extension at 72 °C for 1 min and 40 s, with a final extension at 72 °C for 7 min.

PCR products were visualized by electrophoresis on a 2% agarose gel containing RedSafe (iNtRON Biotechnology, South Korea) and purified using the MEGAquick-spin Plus Total Fragment DNA Purification Kit (iNtRON Biotechnology, South Korea) according to the manufacturer’s protocol. Sanger sequencing was performed by BIONICS Co. (South Korea), and taxonomic identification was confirmed using BLAST searches against the NCBI nr/nt database.

### 2.3 Antimicrobial susceptibility testing

Three to five well-isolated colonies were suspended in 5 mL of cation-adjusted Mueller–Hinton broth (ThermoFisher Scientific) and adjusted to a 0.5 McFarland standard.

A 100-μL aliquot of the suspension was transferred to Brucella broth supplemented with 5% defibrinated sheep blood, 5 μg/mL hemin, and 10 μg/mL vitamin K, yielding a final bacterial concentration of 1 × 10^6^ CFU/mL. The inoculum was dispensed into Sensititre AN02B trays (UniScience, South Korea) and incubated anaerobically at 37 °C until visible growth was observed in the positive control wells. The antimicrobials tested included ampicillin/sulbactam (SAM), amoxicillin/clavulanic acid (AMC), cefotetan (CTT), penicillin (PEN), imipenem (IPM), meropenem (MEM), clindamycin (CLI), cefoxitin (FOX), metronidazole (MTZ), chloramphenicol (CHL), ampicillin (AMP), piperacillin (PIP), tetracycline (TET), mezlocillin (MEZ), and piperacillin/tazobactam (TZP).

The minimum inhibitory concentration (MIC) was defined as the lowest concentration of an antimicrobial that completely inhibited visible bacterial growth. *Bacteroides fragilis* (ATCC 25285) was used as the quality control strain. All assays were performed in triplicate. MIC interpretation was based on the breakpoints established by the European Committee on Antimicrobial Susceptibility Testing (EUCAST) for anaerobic bacteria.

## 3 Results

### 3.1 Resource identification initiative

Overall, 40 *Porphyromonas* spp. were isolated from 30 of the 58 dogs sampled. The species distribution comprised 15 *Porphyromonas gulae*, 11 *Porphyromonas macacae*, eight *Porphyromonas gingivalis*, five *Porphyromonas gingivicanis*, and one *Porphyromonas crevioricanis*. Species identification was consistent across methods, with BLAST identity scores above 98.3% (Supplementary Table S1).

#### 3.1.1 Overall susceptibility patterns

No resistance was observed to SAM, AMC, CTT, FOX, MTZ, CHL, PIP, TET, MEZ, or TZP (Table 1).

**Table 1:** Antimicrobial susceptibility of *Porphyromonas* isolates from the subgingival plaque of dogs with periodontitis.

Antimicrobial	Class	Breakpoint (μg/mL)	Strains (no.)/% resistance				
			*P. gulae* (*n* = 15)	*P. macacae* (*n* = 11)	*P. gingivalis* (*n* = 8)	*P. gingivicanis* (*n* = 5)	*P. creviorcanis* (*n* = 1)
AMP	Penicillin	(≤2)	0	54.5	0	0	100
AMC		(≤8/2)	0	0	0	0	0
SAM		(≤8/4)	0	0	0	0	0
PEN		(≤0.5)	0	36.3	12.5	0	100
PIP		(≤16)	0	0	0	0	0
TZP		(≤32/4)	0	0	0	0	0
MEZ		(≤32)	0	0	0	0	0
CTT	Cephalosporin	(≤16)	0	0	0	0	0
FOX		(≤16)	0	0	0	0	0
IPM	Carbapenem	(≤4)	0	0	37.5	0	0
MEM		(≤8)	0	0	37.5	0	0
CLI	Lincosamide	(≤4)	40	63.6	25	0	0
MTZ	Nitroimidazole	(≤4)	0	0	0	0	0
CHL	Amphenicol	(≤8)	0	0	0	0	0
TET	Tetracycline	(≤4)	0	0	0	0	0

AMP, ampicillin; AMC, amoxicillin/clavulanic acid; CHL, chloramphenicol; CLI, clindamycin; CTT, cefotetan; FOX, cefoxitin; IPM, imipenem; MEM, meropenem; MEZ, mezlocillin; MTZ, metronidazole; PEN, penicillin; PIP, piperacillin; SAM, ampicillin/sulbactam; TET, tetracycline; TZP, piperacillin/tazobactam. Breakpoints (μg/mL), based on EUCAST guidelines: SAM, ≤8/4; AMC, ≤8/2; CTT, ≤16; PEN, ≤0.5; IPM, ≤4; MEM, ≤8; CLI, ≤4; FOX, ≤16; MTZ, ≤4; CHL, ≤8; AMP, ≤2; PIP, ≤16; TET, ≤4; MEZ, ≤32; TZP, ≤32/4.

#### 3.1.2 Penicillin-class agents

Resistance to PEN was detected in six isolates (15%): four *P. macacae* (36.4%), one *P. gingivalis* (12.5%), and the single *P. crevioricanis* isolate (100%).

Resistance to AMP was observed in seven isolates (17.5%): six *P. macacae* (54.5%) and one *P. crevioricanis* (100%).

#### 3.1.3 Carbapenems

Resistance to IPM and MEM was detected in three isolates each (7.5%), all belonging to *P. gingivalis* (37.5%).

#### 3.1.4 Lincosamides

Resistance to CLI was identified in 15 isolates (25%): six *P. gulae* (40%), seven *P. macacae* (63.6%), and two *P. gingivalis* (25%).

Detailed species-specific resistance profiles are provided in Supplementary Tables S2–S6.

## 4 Discussion

In this study, we determined antimicrobial resistance profiles of 40 *Porphyromonas* isolates recovered from 30 of the 58 dogs with periodontal disease. To the best of our knowledge, this is the first report from South Korea to investigate the antimicrobial susceptibility of *Porphyromonas* spp. isolated from the canine dental plaque.

In this study, *Porphyromonas* spp. were isolated from only half of the sampled dogs. This observation likely reflects inter-individual variation in oral microbial communities, differences in disease stage, and host or environmental factors (4). Notably, studies utilizing 16S rRNA sequencing often report higher prevalence rates of *Porphyromonas*, as DNA-based approaches can detect non-cultivable or low-abundance populations (25). In contrast, the culture-based methods employed in the present study may underestimate prevalence due to the fastidious growth requirements of these organisms. This methodological distinction should be considered when interpreting the prevalence data presented here.

CLI is commonly prescribed for the management of bacterial periodontal infections and is frequently used to treat gingivitis, periodontitis, dental root abscesses, and as prophylaxis following oral surgery (26, 27). Among the antimicrobials tested, CLI exhibited the highest resistance rate, in contrast to a previous study by Senorinho et al. (28), which reported no CLI resistance among *P. gulae* and *P. macacae* isolates. Despite this, most Porphyromonas isolates in our study remained susceptible to a broad range of antimicrobials. This may reflect their localization within biofilms, which reduces antimicrobial exposure and the reliance on mechanical debridement (i.e., scaling) as the primary therapy, combined with generally judicious antimicrobial use in veterinary practice (5, 6, 9–11).

In human dentistry, resistance of *Porphyromonas* spp. to clindamycin has been documented in several studies (29, 30), particularly among *P. gingivalis* isolates from patients with periodontal disease, whereas data in companion animals remain scarce. Our study provides region-specific insights that contribute to bridging this gap.

Antimicrobial susceptibility testing is rarely conducted in clinical settings prior to treatment, often resulting in inappropriate or excessive use (31–33). Although antimicrobial susceptibility testing should ideally inform treatment decisions, empirical therapy is sometimes necessary. *Porphyromonas* spp. are fastidious anaerobes that may require up to 2 weeks of incubation to yield visible growth, presenting a challenge in time-sensitive conditions such as sepsis or aspiration pneumonia. In such cases, access to region-specific antimicrobial resistance data is essential to guide empirical therapy until isolate-specific results are available.

In addition to their role in periodontal disease, oral bacteria such as *Porphyromonas* spp. may enter the bloodstream during dental procedures and contribute to systemic conditions, including infective endocarditis (34, 35). Therefore, antimicrobial decisions in veterinary dentistry should consider not only the local oral microbiota but also the potential for systemic sequelae. This highlights the importance of prudent, case-specific antimicrobial use.

A limitation of this study is the absence of detailed clinical metadata for the sampled dogs. Information such as age, sex, breed, disease stage, and prior antimicrobial exposure was not available, as the samples were obtained during routine dental procedures at a collaborating veterinary hospital without access to the patients’ full medical histories. The lack of such data restricted our ability to evaluate potential associations between host characteristics, treatment history, and antimicrobial resistance patterns. Future studies incorporating comprehensive clinical information will be important to better understand risk factors for resistance in canine periodontal pathogens.

Despite these limitations, our findings highlight the clinical importance of considering antimicrobial resistance in periodontal pathogens. Accordingly, veterinarians should consider the use of clindamycin in empirical therapy for canine periodontal infections, whenever feasible, perform culture and susceptibility testing.

## 5 Conclusion

This study confirms the presence of clindamycin-resistant Porphyromonas spp. in dogs with periodontitis in South Korea. While resistance was limited, these findings provide region-specific data to guide empirical therapy and highlight the value of susceptibility testing in veterinary dentistry.

## Data Availability

The original contributions presented in the study are included in the article/Supplementary material, further inquiries can be directed to the corresponding author.
