# Supplementary material for: Antimicrobial susceptibility of Porphyromonas spp. isolated from dogs with periodontal disease in South Korea
Source: Front Vet Sci. 2025 Nov 10;12:1684907. doi: 10.3389/fvets.2025.1684907 (PMC12640808; doi:10.3389/fvets.2025.1684907)
Supplement: Supplementary file 1 [file Data_Sheet_1.docx]

Supplementary Material

# Supplementary Data

**Table S1.** MALDI-TOF result and Corresponding BLAST identity values

| **Species** | **# of Isolates** | **MALDI-TOF result** | **BLAST identity scores** |
| --- | --- | --- | --- |
| ***P. gulae*** | **15** | ***P. gulae*** | **≥98.3%** |
| ***P. macacae*** | **11** | ***P. macacae*** | **≥98.3%** |
| ***P. gingivalis*** | **8** | ***P. gingivalis*** | **≥98.3%** |
| ***P. gingivicanis*** | **5** | ***P. gingivicanis*** | **≥98.3%** |
| ***P. crevioricanis*** | **1** | ***P. crevioricanis*** | **≥98.3%** |

**Table S2.** Minimum inhibitory concentration (MIC) values and antimicrobial resistance profiles of *Porphyromonas gulae* isolates from the subgingival plaque of dogs with periodontitis

| Antimicrobial | Isolate No./MIC (μg/mL) | | | | | | | |
| --- | --- | --- | --- | --- | --- | --- | --- | --- |
|  | 1 | 2 | 3 | 4 | 5 | 6 | 7 | 8 |
| SAM | ≤ 0.5/0.25 | ≤ 0.5/0.25 | ≤ 0.5/0.25 | ≤ 0.5/0.25 | ≤ 0.5/0.25 | ≤ 0.5/0.25 | ≤ 0.5/0.25 | ≤ 0.5/0.25 |
| AMC | ≤ 0.5/0.25 | ≤ 0.5/0.25 | ≤ 0.5/0.25 | ≤ 0.5/0.25 | ≤ 0.5/0.25 | ≤ 0.5/0.25 | ≤ 0.5/0.25 | ≤ 0.5/0.25 |
| CTT | ≤ 4 | ≤ 4 | ≤ 4 | ≤ 4 | ≤ 4 | ≤ 4 | ≤ 4 | ≤ 4 |
| PEN | ≤ 0.06 | ≤ 0.06 | ≤ 0.06 | ≤ 0.06 | ≤ 0.06 | ≤ 0.06 | ≤ 0.06 | ≤ 0.06 |
| IPM | ≤ 0.12 | 1 | 0.5 | ≤ 0.12 | 0.5 | ≤ 0.12 | 1 | 0.5 |
| MEM | ≤ 0.5 | ≤ 0.5 | ≤ 0.5 | ≤ 0.5 | ≤ 0.5 | ≤ 0.5 | ≤ 0.5 | ≤ 0.5 |
| CLI | ≥ 8 | ≤ 0.25 | ≤ 0.25 | ≥ 8 | ≤ 0.25 | ≥ 8 | ≤ 0.25 | ≤ 0.25 |
| FOX | ≤ 1 | ≤ 1 | ≤ 1 | ≤ 1 | ≤ 1 | ≤ 1 | ≤ 1 | ≤ 1 |
| MTZ | ≤ 0.5 | ≤ 0.5 | ≤ 0.5 | ≤ 0.5 | ≤ 0.5 | ≤ 0.5 | ≤ 0.5 | ≤ 0.5 |
| CHL | ≤ 2 | 4 | ≤ 2 | ≤ 2 | ≤ 2 | ≤ 2 | 4 | ≤ 2 |
| AMP | ≤ 0.5 | ≤ 0.5 | ≤ 0.5 | ≤ 0.5 | ≤ 0.5 | ≤ 0.5 | ≤ 0.5 | ≤ 0.5 |
| PIP | ≤ 4 | ≤ 4 | ≤ 4 | ≤ 4 | ≤ 4 | ≤ 4 | ≤ 4 | ≤ 4 |
| TET | ≤ 0.25 | ≤ 0.25 | ≤ 0.25 | ≤ 0.25 | ≤ 0.25 | ≤ 0.25 | ≤ 0.25 | ≤ 0.25 |
| MEZ | ≤ 4 | ≤ 4 | ≤ 4 | ≤ 4 | ≤ 4 | ≤ 4 | ≤ 4 | ≤ 4 |
| TZP | ≤ 0.25/4 | ≤ 0.25/4 | ≤ 0.25/4 | ≤ 0.25/4 | ≤ 0.25/4 | ≤ 0.25/4 | ≤ 0.25/4 | ≤ 0.25/4 |

| Antimicrobial | Isolate No./MIC (μg/mL) | | | | | | |
| --- | --- | --- | --- | --- | --- | --- | --- |
|  | 9 | 10 | 11 | 12 | 13 | 14 | 15 |
| SAM | ≤ 0.5/0.25 | ≤ 0.5/0.25 | ≤ 0.5/0.25 | ≤ 0.5/0.25 | ≤ 0.5/0.25 | ≤ 0.5/0.25 | ≤ 0.5/0.25 |
| AMC | ≤ 0.5/0.25 | ≤ 0.5/0.25 | ≤ 0.5/0.25 | ≤ 0.5/0.25 | ≤ 0.5/0.25 | ≤ 0.5/0.25 | ≤ 0.5/0.25 |
| CTT | ≤ 4 | ≤ 4 | ≤ 4 | ≤ 4 | ≤ 4 | ≤ 4 | ≤ 4 |
| PEN | ≤ 0.06 | ≤ 0.06 | ≤ 0.06 | ≤ 0.06 | ≤ 0.06 | ≤ 0.06 | ≤ 0.06 |
| IPM | 1 | 0.5 | ≤ 0.12 | 1 | 0.5 | ≤ 0.12 | 0.5 |
| MEM | ≤ 0.5 | ≤ 0.5 | ≤ 0.5 | ≤ 0.5 | ≤ 0.5 | ≤ 0.5 | ≤ 0.5 |
| CLI | ≤ 0.25 | ≤ 0.25 | ≥ 8 | ≤ 0.25 | ≤ 0.25 | ≥ 8 | ≥ 8 |
| FOX | ≤ 1 | ≤ 1 | ≤ 1 | ≤ 1 | ≤ 1 | ≤ 1 | ≤ 1 |
| MTZ | ≤ 0.5 | ≤ 0.5 | ≤ 0.5 | ≤ 0.5 | ≤ 0.5 | ≤ 0.5 | ≤ 0.5 |
| CHL | 4 | ≤ 2 | ≤ 2 | 4 | ≤ 2 | ≤ 2 | ≤ 2 |
| AMP | ≤ 0.5 | ≤ 0.5 | ≤ 0.5 | ≤ 0.5 | ≤ 0.5 | ≤ 0.5 | ≤ 0.5 |
| PIP | ≤ 4 | ≤ 4 | ≤ 4 | ≤ 4 | ≤ 4 | ≤ 4 | ≤ 4 |
| TET | ≤ 0.25 | ≤ 0.25 | ≤ 0.25 | ≤ 0.25 | ≤ 0.25 | ≤ 0.25 | ≤ 0.25 |
| MEZ | ≤ 4 | ≤ 4 | ≤ 4 | ≤ 4 | ≤ 4 | ≤ 4 | ≤ 4 |
| TZP | ≤ 0.25/4 | ≤ 0.25/4 | ≤ 0.25/4 | ≤ 0.25/4 | ≤ 0.25/4 | ≤ 0.25/4 | ≤ 0.25/4 |

*Red text indicates antimicrobial resistance.* **Abbreviations**: AMP: Ampicillin, AMC: Amoxicillin/clavulanic acid, CHL: Chloramphenicol, CLI: Clindamycin, CTT: Cefotetan, FOX: Cefoxitin, IPM: Imipenem, MEM: Meropenem, MEZ: Mezlocillin, MTZ: Metronidazole, PEN: Penicillin, PIP: Piperacillin, SAM: Ampicillin/sulbactam, TET: Tetracycline, TZP: Piperacillin/tazobactam. **Breakpoints** (μg/mL), as per EUCAST guidelines: SAM, ≤ 8/4; AMC, ≤ 8/2; CTT, ≤ 16; PEN, ≤ 0.5; IPM, ≤ 4; MEM, ≤ 8; CLI, ≤ 4; FOX, ≤ 16; MTZ, ≤ 4; CHL, ≤ 8; AMP, ≤ 2; PIP, ≤ 16; TET, ≤ 4; MEZ, ≤ 32; TZP, ≤ 32/4.

**Table S3.** Minimum inhibitory concentration (MIC) values and antimicrobial resistance profiles of *Porphyromonas macacae* isolates from the subgingival plaque of dogs with periodontitis

| Antimicrobial | Isolate No./MIC (μg/mL) | | | | | | | | |  | |  |  | |  |
| --- | --- | --- | --- | --- | --- | --- | --- | --- | --- | --- | --- | --- | --- | --- | --- |
|  | 1 | 2 | 3 | 4 | 5 | 6 | 7 | 8 | 9 | | 10 | | | 11 |  |
| SAM | ≤ 0.5/0.25 | ≤ 0.5/0.25 | ≤ 0.5/0.25 | ≤ 0.5/0.25 | ≤ 0.5/0.25 | ≤ 0.5/0.25 | ≤ 0.5/0.25 | ≤ 0.5/0.25 | ≤ 0.5/0.25 | | ≤ 0.5/0.25 | | | ≤ 0.5/0.25 |  |
|  |  |  |  |  |  |  |  |  |  | |  | | |  |  |
| AMC | ≤ 0.5/0.25 | ≤ 0.5/0.25 | ≤ 0.5/0.25 | ≤ 0.5/0.25 | ≤ 0.5/0.25 | ≤ 0.5/0.25 | ≤ 0.5/0.25 | ≤ 0.5/0.25 | ≤ 0.5/0.25 | | ≤ 0.5/0.25 | | | ≤ 0.5/0.25 |  |
| CTT | ≤ 4 | ≤ 4 | ≤ 4 | ≤ 4 | ≤ 4 | ≤ 4 | ≤ 4 | ≤ 4 | ≤ 4 | | ≤ 4 | | | ≤ 4 |  |
| PEN | 4 | ≥ 8 | 2 | 1 | ≤ 0.06 | ≤ 0.06 | ≤ 0.06 | ≤ 0.06 | ≤ 0.06 | | ≤ 0.06 | | | ≤ 0.06 |  |
| IPM | 1 | ≤ 0.12 | 1 | ≤ 0.12 | ≤ 0.12 | ≤ 0.12 | ≤ 0.12 | ≤ 0.12 | ≤ 0.12 | | ≤ 0.12 | | | ≤ 0.12 |  |
| MEM | ≤ 0.5 | ≤ 0.5 | ≤ 0.5 | ≤ 0.5 | ≤ 0.5 | ≤ 0.5 | ≤ 0.5 | ≤ 0.5 | ≤ 0.5 | | ≤ 0.5 | | | ≤ 0.5 |  |
| CLI | ≥ 8 | ≤ 0.25 | ≤ 0.25 | ≥ 8 | ≤ 0.25 | ≥ 8 | ≥ 8 | ≥ 8 | ≥ 8 | | ≤ 0.25 | | | ≥ 8 |  |
| FOX | 2 | ≤ 1 | ≤ 1 | ≤ 1 | ≤ 1 | ≤ 1 | ≤ 1 | ≤ 1 | ≤ 1 | | ≤ 1 | | | ≤ 1 |  |
| MTZ | ≤ 0.5 | ≤ 0.5 | ≤ 0.5 | ≤ 0.5 | ≤ 0.5 | ≤ 0.5 | ≤ 0.5 | ≤ 0.5 | ≤ 0.5 | | ≤ 0.5 | | | ≤ 0.5 |  |
| CHL | 4 | 4 | ≤ 2 | ≤ 2 | ≤ 2 | ≤ 2 | ≤ 2 | ≤ 2 | ≤ 2 | | ≤ 2 | | | ≤ 2 |  |
| AMP | 2 | ≥ 16 | 2 | 1 | 1 | 1 | ≤ 0.5 | 1 | ≥ 16 | | 2 | | | ≥ 16 |  |
| PIP | ≤ 4 | ≤ 4 | ≤ 4 | ≤ 4 | ≤ 4 | ≤ 4 | ≤ 4 | ≤ 4 | ≤ 4 | | ≤ 4 | | | ≤ 4 |  |
| TET | ≤ 0.25 | ≤ 0.25 | ≤ 0.25 | ≤ 0.25 | ≤ 0.25 | ≤ 0.25 | ≤ 0.25 | ≤ 0.25 | ≤ 0.25 | | ≤ 0.25 | | | ≤ 0.25 |  |
| MEZ | ≤ 4 | ≤ 4 | ≤ 4 | ≤ 4 | ≤ 4 | ≤ 4 | ≤ 4 | ≤ 4 | ≤ 4 | | ≤ 4 | | | ≤ 4 |  |
| TZP | ≤ 0.25/4 | ≤ 0.25/4 | ≤ 0.25/4 | ≤ 0.25/4 | ≤ 0.25/4 | ≤ 0.25/4 | ≤ 0.25/4 | ≤ 0.25/4 | ≤ 0.25/4 | | ≤ 0.25/4 | | | ≤ 0.25/4 |  |

*Red text indicates antimicrobial resistance.* **Abbreviations**: AMP: Ampicillin, AMC: Amoxicillin/clavulanic acid, CHL: Chloramphenicol, CLI: Clindamycin, CTT: Cefotetan, FOX: Cefoxitin, IPM: Imipenem, MEM: Meropenem, MEZ: Mezlocillin, MTZ: Metronidazole, PEN: Penicillin, PIP: Piperacillin, SAM: Ampicillin/sulbactam, TET: Tetracycline, TZP: Piperacillin/tazobactam. **Breakpoints** (μg/mL), according to EUCAST: SAM, ≤ 8/4; AMC, ≤ 8/2; CTT, ≤ 16; PEN, ≤ 0.5; IPM, ≤ 4; MEM, ≤ 8; CLI, ≤ 4; FOX, ≤ 16; MTZ, ≤ 4; CHL, ≤ 8; AMP, ≤ 2; PIP, ≤ 16; TET, ≤ 4; MEZ, ≤ 32; TZP, ≤ 32/4.

| Antimicrobial | Isolate No./MIC (μg/mL) | | | | | | | |
| --- | --- | --- | --- | --- | --- | --- | --- | --- |
|  | 1 | 2 | 3 | 4 | 5 | 6 | 7 | 8 |
| SAM | ≤ 0.5/0.25 | ≤ 0.5/0.25 | ≤ 0.5/0.25 | ≤ 0.5/0.25 | ≤ 0.5/0.25 | ≤ 0.5/0.25 | ≤ 0.5/0.25 | ≤ 0.5/0.25 |
| AMC | ≤ 0.5/0.25 | ≤ 0.5/0.25 | ≤ 0.5/0.25 | ≤ 0.5/0.25 | ≤ 0.5/0.25 | ≤ 0.5/0.25 | ≤ 0.5/0.25 | ≤ 0.5/0.25 |
| CTT | ≤ 4 | ≤ 4 | ≤ 4 | ≤ 4 | ≤ 4 | ≤ 4 | ≤ 4 | ≤ 4 |
| PEN | ≤ 0.06 | 1 | ≤ 0.06 | ≤ 0.06 | ≤ 0.06 | ≤ 0.06 | ≤ 0.06 | ≤ 0.06 |
| IPM | ≤ 0.12 | ≥ 8 | 1 | 1 | ≤ 0.12 | ≥ 8 | 0.5 | ≥ 8 |
| MEM | ≤ 0.5 | ≤ 0.5 | ≤ 0.5 | ≤ 0.5 | ≤ 0.5 | ≥ 8 | 8 | ≥ 8 |
| CLI | ≤ 0.25 | ≤ 0.25 | ≥ 8 | ≥ 8 | ≤ 0.25 | ≤ 0.25 | ≤ 0.25 | ≤ 0.25 |
| FOX | ≤ 1 | ≤ 1 | ≤ 1 | ≤ 1 | ≤ 1 | ≤ 1 | ≤ 1 | ≤ 1 |
| MTZ | ≤ 0.5 | ≤ 0.5 | ≤ 0.5 | ≤ 0.5 | ≤ 0.5 | ≤ 0.5 | ≤ 0.5 | ≤ 0.5 |
| CHL | 4 | ≤ 2 | 4 | ≤ 2 | ≤ 2 | ≤ 2 | ≤ 2 | ≤ 2 |
| AMP | ≤ 0.5 | ≤ 0.5 | ≤ 0.5 | ≤ 0.5 | ≤ 0.5 | ≤ 0.5 | ≤ 0.5 | ≤ 0.5 |
| PIP | ≤ 4 | ≤ 4 | ≤ 4 | ≤ 4 | ≤ 4 | ≤ 4 | ≤ 4 | ≤ 4 |
| TET | ≤ 0.25 | ≤ 0.25 | ≤ 0.25 | ≤ 0.25 | ≤ 0.25 | ≤ 0.25 | ≤ 0.25 | ≤ 0.25 |
| MEZ | ≤ 4 | ≤ 4 | ≤ 4 | ≤ 4 | ≤ 4 | ≤ 4 | ≤ 4 | ≤ 4 |
| TZP | ≤ 0.25/4 | ≤ 0.25/4 | ≤ 0.25/4 | ≤ 0.25/4 | ≤ 0.25/4 | ≤ 0.25/4 | ≤ 0.25/4 | ≤ 0.25/4 |

**Table S4.** Minimum inhibitory concentration (MIC) values and antimicrobial resistance profiles of *Porphyromonas gingivalis* isolates from the subgingival plaque of dogs with periodontitis

*Red text indicates antimicrobial resistance.* **Abbreviations**: AMP: Ampicillin, AMC: Amoxicillin/clavulanic acid, CHL: Chloramphenicol, CLI: Clindamycin, CTT: Cefotetan, FOX: Cefoxitin, IPM: Imipenem, MEM: Meropenem, MEZ: Mezlocillin, MTZ: Metronidazole, PEN: Penicillin, PIP: Piperacillin, SAM: Ampicillin/sulbactam, TET: Tetracycline, TZP: Piperacillin/tazobactam. **Breakpoints** (μg/mL), according to EUCAST: SAM, ≤ 8/4; AMC, ≤ 8/2; CTT, ≤ 16; PEN, ≤ 0.5; IPM, ≤ 4; MEM, ≤ 8; CLI, ≤ 4; FOX, ≤ 16; MTZ, ≤ 4; CHL, ≤ 8; AMP, ≤ 2; PIP, ≤ 16; TET, ≤ 4; MEZ, ≤ 32; TZP, ≤ 32/4.

| Antimicrobial | Isolate No./MIC (μg/mL) | | | | |
| --- | --- | --- | --- | --- | --- |
|  | 1 | 2 | 3 | 4 | 5 |
| SAM | ≤ 0.5/0.25 | ≤ 0.5/0.25 | ≤ 0.5/0.25 | ≤ 0.5/0.25 | ≤ 0.5/0.25 |
| AMC | ≤ 0.5/0.25 | ≤ 0.5/0.25 | ≤ 0.5/0.25 | ≤ 0.5/0.25 | ≤ 0.5/0.25 |
| CTT | ≤ 4 | ≤ 4 | ≤ 4 | ≤ 4 | ≤ 4 |
| PEN | ≤ 0.06 | ≤ 0.06 | ≤ 0.06 | ≤ 06 | ≤ 0.06 |
| IPM | ≤ 0.12 | ≤ 0.12 | ≤ 0.12 | ≤ 0.12 | ≤ 0.12 |
| MEM | ≤ 0.5 | ≤ 0.5 | ≤ 0.5 | ≤ 0.5 | ≤ 0.5 |
| CLI | ≤ 0.25 | ≤ 0.25 | ≤ 0.25 | ≤ 0.25 | ≤ 0.25 |
| FOX | ≤ 1 | ≤ 1 | ≤ 1 | ≤ 1 | ≤ 1 |
| MTZ | ≤ 0.5 | ≤ 0.5 | ≤ 0.5 | ≤ 0.5 | ≤ 0.5 |
| CHL | ≤ 2 | ≤ 2 | ≤ 2 | ≤ 2 | ≤ 2 |
| AMP | 1 | 1 | 1 | 1 | 1 |
| PIP | ≤ 4 | ≤ 4 | ≤ 4 | ≤ 4 | ≤ 4 |
| TET | ≤ 0.25 | ≤ 0.25 | ≤ 0.25 | ≤ 0.25 | ≤ 0.25 |
| MEZ | ≤ 4 | ≤ 4 | ≤ 4 | ≤ 4 | ≤ 4 |
| TZP | ≤ 0.25/4 | ≤ 0.25/4 | ≤ 0.25/4 | ≤ 0.25/4 | ≤ 0.25/4 |

**Table S5.** Minimum inhibitory concentration (MIC) values and antimicrobial resistance profiles of *Porphyromonas gingivicanis* isolates from the subgingival plaque of dogs with periodontitis

*Red text indicates antimicrobial resistance.* **Abbreviations**: AMP: Ampicillin, AMC: Amoxicillin/clavulanic acid, CHL: Chloramphenicol, CLI: Clindamycin, CTT: Cefotetan, FOX: Cefoxitin, IPM: Imipenem, MEM: Meropenem, MEZ: Mezlocillin, MTZ: Metronidazole, PEN: Penicillin, PIP: Piperacillin, SAM: Ampicillin/sulbactam, TET: Tetracycline, TZP: Piperacillin/tazobactam. **Breakpoints** (μg/mL), based on EUCAST guidelines: SAM, ≤ 8/4; AMC, ≤ 8/2; CTT, ≤ 16; PEN, ≤ 0.5; IPM, ≤ 4; MEM, ≤ 8; CLI, ≤ 4; FOX, ≤ 16; MTZ, ≤ 4; CHL, ≤ 8; AMP, ≤ 2; PIP, ≤ 16; TET, ≤ 4; MEZ, ≤ 32; TZP, ≤ 32/4.

| Antimicrobial | Isolate No./MIC (μg/mL) |
| --- | --- |
|  | 1 |
| SAM | ≤ 0.5/0.25 |
| AMC | ≤ 0.5/0.25 |
| CTT | ≤ 4 |
| PEN | 4 |
| IPM | ≤ 0.12 |
| MEM | ≤ 0.5 |
| CLI | ≤ 0.25 |
| FOX | 8 |
| MTZ | ≤ 0.5 |
| CHL | ≤ 2 |
| AMP | 8 |
| PIP | ≤ 4 |
| TET | ≤ 0.25 |
| MEZ | ≤ 4 |
| TZP | ≤ 0.25/4 |

**Table S5.** Minimum inhibitory concentration (MIC) values and antimicrobial resistance profile of *Porphyromonas crevioricanis* isolated from the subgingival plaque of a dog with periodontitis

*Red text indicates antimicrobial resistance.* **Abbreviations**: AMP: Ampicillin, AMC: Amoxicillin/clavulanic acid, CHL: Chloramphenicol, CLI: Clindamycin, CTT: Cefotetan, FOX: Cefoxitin, IPM: Imipenem, MEM: Meropenem, MEZ: Mezlocillin, MTZ: Metronidazole, PEN: Penicillin, PIP: Piperacillin, SAM: Ampicillin/sulbactam, TET: Tetracycline, TZP: Piperacillin/tazobactam. **Breakpoints** (μg/mL), based on EUCAST guidelines: SAM, ≤ 8/4; AMC, ≤ 8/2; CTT, ≤ 16; PEN, ≤ 0.5; IPM, ≤ 4; MEM, ≤ 8; CLI, ≤ 4; FOX, ≤ 16; MTZ, ≤ 4; CHL, ≤ 8; AMP, ≤ 2; PIP, ≤ 16; TET, ≤ 4; MEZ, ≤ 32; TZP
